# Changes in Clinical Manifestations Due to AFLD Retyping Based on the New MAFLD Criteria: An Observational Study Based on the National Inpatient Sample Database

**DOI:** 10.3390/diagnostics13030488

**Published:** 2023-01-29

**Authors:** Xiaoshan Feng, Ruirui Xuan, Yingchun Dong, Xiaoqin Wu, Yiping Cheng, Zinuo Yuan, Hang Dong, Junming Han, Fang Zhong, Jiajun Zhao, Xiude Fan

**Affiliations:** 1 Department of Endocrinology, Shandong Provincial Hospital Affiliated to Shandong First Medical University, Jinan 250021, China; 2 Shandong Clinical Research Center of Diabetes and Metabolic Diseases, Jinan 250021, China; 3 Shandong Institute of Endocrine and Metabolic Diseases, Jinan 250021, China; 4 Shandong Engineering Laboratory of Prevention and Control for Endocrine and Metabolic Diseases, Jinan 250021, China; 5 Shandong Engineering Research Center of Stem Cell and Gene Therapy for Endocrine and Metabolic Diseases, Jinan 250021, China; 6 Northern Ohio Alcohol Center, Department of Inflammation and Immunity, Cleveland Clinic, Cleveland, OH 44196, USA

**Keywords:** alcohol fatty liver disease, metabolic-associated fatty liver disease, comorbidities, organ failure, prevalence rate, odds ratio

## Abstract

(1) Background: As the introduction of “positive” diagnostic criteria for metabolic dysfunction-associated fatty liver disease (MAFLD) does not exclude alcohol consumption, some patients originally diagnosed with alcoholic fatty liver disease (AFLD) may be diagnosed with dual- etiology fatty liver disease (AFLD&MAFLD), which requires us to urgently explore the impact of the changes in this classification of AFLD on clinical manifestations. (2) Methods: Utilizing data from the Nationwide Inpatient Sample database 2016–2018, a total of 9269 participants with AFLD were selected. With the definition of MAFLD, these patients were further categorized into two groups: single AFLD and AFLD&MAFLD. The primary outcome was the risk of comorbidities and organ failures. The secondary outcomes were the length of stay, total charges, and in-hospital all-cause mortality. (3) Results: The patients with AFLD&MAFLD were older, were predominantly male, and had more comorbidities and organ failures compared to the patients with AFLD. These comorbidities included coronary atherosclerosis, myocardial infarction, cerebrovascular disease, arrhythmia, asthma, chronic obstructive pulmonary disease, and chronic kidney disease (all *p* values < 0.05). The patients with AFLD&MAFLD were more likely to develop acute and chronic heart and/or kidney failures than those with single AFLD (all *p* < 0.05). The length of stay and total charges of the patients in the AFLD&MAFLD group were greater than the single AFLD group (*p* = 0.029 and *p* < 0.001, respectively). No significant difference in all-cause mortality was observed. (4) Conclusions: The patients with AFLD&MAFLD have more comorbidities and organ failures, longer hospital stays, and higher hospitalization costs than the patients with single AFLD. Hence, patients with dual-etiology fatty liver disease deserve more attention from clinical staff during treatment.

## 1. Introduction

A “positive criterion” for diagnosing metabolic dysfunction-associated fatty liver disease (MAFLD) was proposed in 2020, given its pathogenesis and increasing prevalence [1]. The diagnostic criteria for MAFLD are based on the evidence of hepatic steatosis and the coexistence of overweight or obesity, type 2 diabetes mellitus (T2DM), or metabolic dysregulation [1,2]. The establishment of the “positive criterion” for MAFLD, including alcohol consumption, does not require the exclusion of other chronic liver diseases, including alcohol consumption, as in the previous diagnosis of non-alcoholic fatty liver disease (NAFLD), which may result in individuals with alcoholic fatty liver disease (AFLD) with metabolic dysfunction being classified as dual-etiology fatty liver disease [3]. As an initial hepatic embodiment of alcoholic liver disease [4], the incidence of AFLD has increased [5]. Moreover, the incidence of metabolic dysregulation-related diseases has even so increased [6,7,8]. Therefore, we aimed to explore the influence of the changes in the classification of AFLD on clinical manifestations.

**Figure 1:**
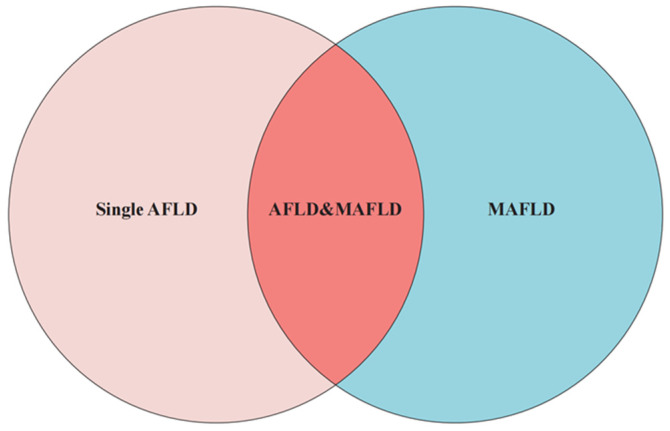
Explanatory chart of single AFLD and AFLD&MAFLD. We define concordant AFLD&MAFLD (dual-etiology fatty liver disease) as individuals who meet both AFLD and MAFLD definitions. In terms of discordant groups, individuals who met the definition of AFLD rather than MAFLD are defined as single AFLD. The size of the graphic area does not represent the actual proportion of people. Abbreviation: AFLD, alcoholic fatty liver disease; AFLD&MAFLD, alcoholic fatty liver disease and metabolic-associated fatty liver disease (dual-etiology fatty liver disease).

Fatty liver disease includes AFLD and NAFLD. The prevalence of AFLD in the United States was as high as 4.7% in 2016, and the rapid progression of AFLD poses a huge health and economic burden [9]. Earlier studies have found that AFLD may progress more rapidly to cirrhosis when combined with obesity and metabolic abnormalities [10]. After the definition of MAFLD was proposed, significant differences in the occurrence, development, and outcome of the disease between patients with MAFLD and NAFLD emerged. MAFLD covers more individuals with health risks than NAFLD [11] and identifies a significant group of people with more comorbidities and worse prognoses than those with NAFLD alone [12]. According to the Third National Health and Nutrition Examination Survey in the United States, patients with MAFLD have a higher risk of all-cause death than patients with NAFLD [11]. Interventions in lifestyle, diet, and behavioral therapies are crucial for the occurrence and treatment of NAFLD [13]. However, with the introduction of the concept of MAFLD, the differences in disease characteristics and progression between dual-etiology fatty liver disease (AFLD&MAFLD) and single AFLD are still largely unknown and worth further exploration (Figure 1).

We conducted an observational study based on the Nationwide Inpatient Sample (NIS) database to compare the risk of comorbidities and organ failures, length of stay, total hospital costs, and in-hospital all-cause mortality between the dual-etiology group (AFLD&MAFLD) and single AFLD group.

## 2. Materials and Methods

### 2.1. Study Population and Study Design

As a part of the family databases and software tools conducted for the Healthcare Cost and Utilization Project (HCUP), the National Inpatient Sample (NIS) is the largest publicly available all-payer inpatient healthcare database designed to produce U.S. regional and national estimates of inpatient utilization, access, cost, quality, and outcomes. Primary and secondary hospitalization diagnoses, patient demographics, length of stay, discharge status, in-hospital mortality, and severity/comorbidity measures are available in the NIS database.

In this study, we obtained data from the NIS database from 1 January 2016 to 31 December 2018. This study was based on the STROBE [14] reporting guidelines and abided by the United States Agency for Healthcare Research and Quality’s Healthcare Cost and Utilization Project Data Use Agreement; it was exempt from research ethics board review. We included adults with discharge diagnoses of AFLD (including but not limited to the reason for admission) from the NIS database. We excluded (1) patients aged < 18; (2) patients combined with hepatocellular carcinoma, hepatic cirrhosis, virus hepatitis, autoimmune liver disease, and Wilson’s disease; (3) patients with pregnancy; and (4) patients missing data for baseline characteristics for analysis. The details of the inclusion and exclusion criteria and the International Classification of Diseases, 10th Revision (ICD-10) codes used are presented in Supplementary Tables S1 and S2, respectively.

### 2.2. Data Collection

We collected the following patients’ characteristics: age, sex, race, length of stay (LOS), total charges, and admission type (elective or non-elective). We also collected data on comorbidities, which included coronary atherosclerosis, acute myocardial infarction, cerebrovascular disease, peripheral vascular disease, arrhythmia, asthma, chronic obstructive pulmonary disease, and chronic kidney disease. Besides that, we collected data on chronic and acute organ failures of the heart, lung, liver, and kidney. All the ICD-10 codes used to identify comorbidities and organ failures are shown in Supplementary Table S2.

### 2.3. Definition of AFLD&MAFLD and Single AFLD

According to the definition, MAFLD was diagnosed when hepatic steatosis (detected by sonography, computed tomography, and magnetic resonance imaging (MRI) of the liver or serum markers or biopsy) combined with at least one of the following conditions: overweight or obesity, presence of type 2 diabetes mellitus (T2DM), or evidence of metabolic dysregulation. Metabolic dysregulation was defined by the presence of at least two of the following metabolic risk abnormalities: (1) blood pressure at 130/85 mm Hg or specific drug treatment; (2) plasma triglycerides at 150 mg/dL (1.70 mmol/L) or specific drug treatment; (3) plasma high-density lipoprotein cholesterol at <40 mg/dL (<1.0 mmol/L) for men and <50 mg/dL (<1.3 mmol/L) for women or specific drug treatment; (4) prediabetes (fasting glucose levels at 100–125 mg/dL [5.6–6.9 mmol/L] or hemoglobin A1c of 5.7%–6.4% [39–47 mmol/mol]); (5) Homeostatic Model Assessment for Insulin Resistance score of 2.5; and (6) plasma high-sensitivity C-reactive protein level at >2 mg/L [1,15]. However, the above metabolism-related explicit laboratory values are not available in the NIS database. So, we used the following diagnoses based on the ICD10 codes to confirm metabolic dysregulation: (1) overweight or obesity; (2) T2DM; (3) hyperlipidemia: high serum triglyceride (TGs) levels or high high-density lipoprotein (HDL)-cholesterol levels, etc.; (4) hypertension; (5) hyperglycemia; (6) prediabetes; and (7) elevated high-sensitivity C-reactive protein. AFLD was defined as the presence of fatty liver disease and excessive alcohol use (alcohol intake of 30 g/d for men and 20 g/d for women) or elevation in liver enzymes (aspartate aminotransferase (AST)>alanine aminotransferase (ALT)); serum bilirubin at <3 mg/dL; and the absence of other causes of liver disease [16]. All those patients with discharge diagnoses of AFLD were then divided into two groups. AFLD combined with MAFLD (as confirmed by related diagnoses mentioned above) was called dual-etiology fatty liver disease (AFLD&MAFLD), and the rest of the patients with AFLD were divided into a single AFLD group.

### 2.4. Outcomes

Our primary outcomes were the incidence rate and risks of comorbidities and organ failures. Comorbidities included coronary atherosclerosis, acute myocardial infarction, cerebrovascular disease, peripheral vascular disease, arrhythmia, asthma, chronic obstructive pulmonary disease, and chronic kidney disease. Organ failures included chronic and acute failures of the heart, respiratory system, kidney, and liver. In our analyses of the secondary outcomes, we also assessed the differences in length of hospital stay, total charges, and all-cause mortality during hospitalization between the patients with AFLD&MAFLD and those with single AFLD.

### 2.5. Statistical Analysis

The baseline characteristics of the participants were reported for demographic and clinical characteristics of interest using counts and percentages. Continuous variables were presented as means ± standard deviation (SDs). When comparing the differences in the continuous variables between the two groups, we used the Student’s *t*-test and the Mann–Whitney U test separately, depending on if the continuous variables were distributed normally. Categorical variables were presented as numbers (proportions) and compared using the Chi-squared test.

For the primary and secondary outcomes of our study, within the single AFLD and AFLD&MAFLD groups, we calculated the prevalence rates and odds ratio of comorbidities, organ failure, and all-cause in-hospital mortality. The odds ratios and 95% confidence intervals (CIs) for comorbidities and organ failure were estimated using binary regression analysis. Adjusted odds ratios (AOR) (95% CIs) were used for age, sex, race, smoking habit, comorbidities (using other comorbidities correspondingly when conducting specific morbidity analysis), and organ failures (using other organ failures correspondingly when conducting specific organ failure analysis). We took the LOS ≥ 9 days and total charges ≥50,000 dollars as the outcomes and further conducted a binary regression analysis to obtain the odds ratios (adjusted for age, sex, race, and smoking habit). We considered a two-sided *p* value < 0.05 as significant and used a confidence level of 95% for all intervals, unless otherwise noted. The SPSS 26.0 Statistics software (SPSS Inc., Chicago, IL, USA) was used to analyze the data.

## 3. Results

### 3.1. Demographic and Clinical Characteristics of Patients in the Two AFLD Groups

**Figure 2:**
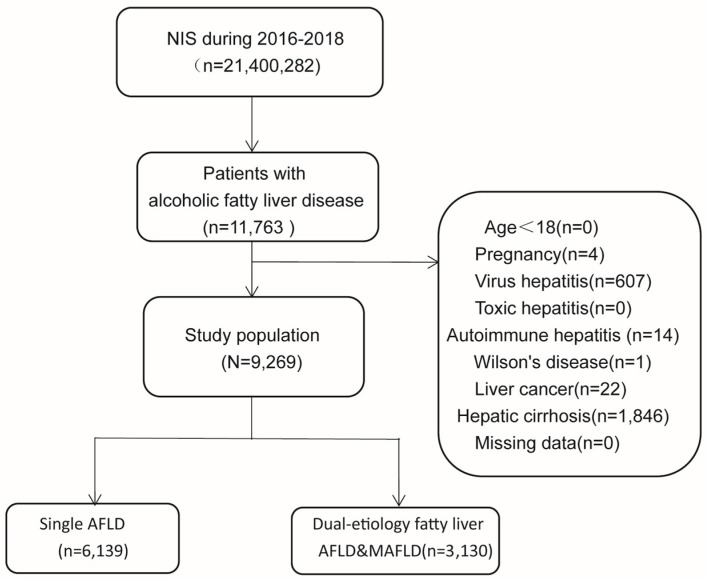
Flow chart of study. Abbreviation: AFLD, alcoholic fatty liver disease; AFLD&MAFLD, alcoholic fatty liver disease and metabolic-associated fatty liver disease (dual–etiology fatty liver disease).

**Figure 3:**
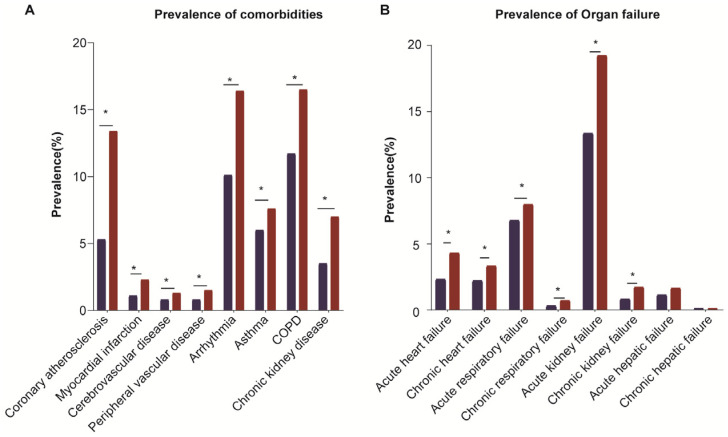
Prevalence of comorbidities and organ failure in patients with single AFLD or AFLD&MAFLD. (**A**) Prevalence of comorbidities, including coronary atherosclerosis, myocardial infarction, arrythmia, asthma, chronic obstructive pulmonary disease (COPD), chronic kidney disease, and peripheral vascular disease, in patients with single AFLD and AFLD&MAFLD. (**B**) Prevalence of organ failure, including acute heart failure, chronic heart failure, acute respiratory failure, chronic respiratory failure, acute hepatic failure, and chronic hepatic failure, in patients with single AFLD and AFLD&MAFLD. * *p* < 0.05 was deemed statistically significant.

A total of 9269 patients with AFLD were included in the NIS database (Figure 2. Flow chart). Based on the MAFLD diagnostic criteria, 6139 patients (66.23%) had single AFLD and 3130 (33.77%) had dual-etiology fatty liver disease (AFLD&MAFLD). The patients with AFLD&MAFLD were older (mean age 52.7 ± 14.5 vs. 47.7 ± 12.9 years, *p* < 0.001), and the proportion of men was higher (74.2% vs. 67.7%, *p* < 0.001) compared to the patients with single AFLD. The patients with AFLD&MAFLD had a higher prevalence of serious comorbidities, such as coronary atherosclerosis (13.4% vs. 5.3%, *p* < 0.001), myocardial infarction (2.3% vs. 1.1%, *p* < 0.001), arrhythmia (16.4% vs. 10.1%, *p* < 0.001), and cerebrovascular disease (1.3% vs. 0.8%, *p* = 0.034), compared to the patients with single AFLD. Furthermore, the prevalence of hypertension, chronic obstructive pulmonary disease (COPD), and chronic kidney disease (CKD) was higher in the AFLD&MAFLD group than in the single AFLD group (all *p* values < 0.001) (Table 1, Figure 3). In addition, the risk of acute (4.3% vs. 2.3%, *p* < 0.001) and chronic heart failures (3.3% vs. 2.2%, *p* = 0.002), acute (7.96% vs. 6.76%, *p* = 0.035) and chronic respiratory failures (0.7% vs. 0.3%, *p* = 0.012), and acute (19.23% vs. 13.34%, *p* < 0.001) and chronic renal failures (1.7% vs. 0.8%, *p* < 0.001) was higher in the AFLD& MAFLD group than in the single AFLD group. However, the prevalence of acute and chronic liver failures had no significant difference between the AFLD&MAFLD and single AFLD groups (all *p* values > 0.05, Figure 3).

### 3.2. High Risk of Comorbidities and Organ Failure in the AFLD&MAFLD Group

We adjusted for potential confounders, such as age, sex, race, smoking habits, and other comorbidities (including coronary atherosclerosis, myocardial infarction, cerebrovascular disease, peripheral vascular disease, arrhythmia, asthma, COPD, chronic kidney disease, acute and chronic organ failure of the heart, lung, liver, and kidney), and when we analyzed a specific disease, we excluded the other diseases. The AFLD&MAFLD group had a higher risk of myocardial infarction (adjusted odds ratio (AOR) = 1.51, 95% CI: 1.06–2.16, *p* = 0.022) and coronary atherosclerosis (AOR = 1.99, 95% CI: 1.70–2.35, *p* < 0.001) than the single AFLD group (Figure 3, Supplementary Table S3). Similar results were found for other comorbidities, with a significantly higher risk of arrhythmia (AOR = 1.33, 95% CI: 1.16–1.52, *p* < 0.001), asthma (AOR =1.40, 95% CI: 1.17–1.68, *p* < 0.001), COPD (AOR = 1.19, 95% CI: 1.02–1.38, *p* = 0.023), and CKD (AOR = 1.36, 95% CI:1.06–1.74, *p* = 0.014) in the AFLD&MAFLD group than in the AFLD group. However, the risk of cerebrovascular disease and peripheral vascular disease did not differ significantly between the AFLD&MAFLD and single AFLD groups (Supplementary Table S3).

**Figure 4:**
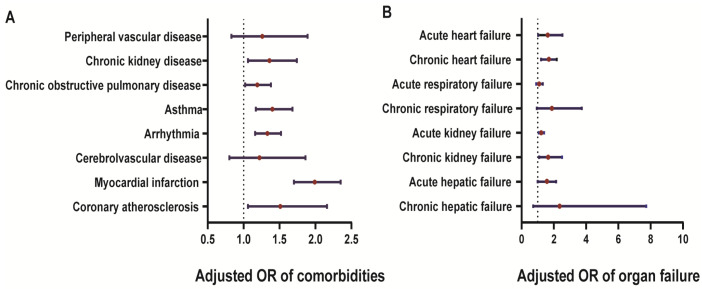
Adjusted odds ratios for comorbidities and organ failure when comparing AFLD&MAFLD to single AFLD. (**A**) Risk of comorbidities, including coronary atherosclerosis, myocardial infarction, arrythmia, asthma, chronic obstructive pulmonary disease (COPD), chronic kidney disease, and peripheral vascular disease, in patients with AFLD&MAFLD. (**B**) Risk of organ failure, including acute heart failure, chronic heart failure, acute respiratory failure, chronic respiratory failure, acute hepatic failure, and chronic hepatic failure, in patients with AFLD&MAFLD.

The risk of acute and chronic heart failures in the patients with AFLD&MAFLD was 1.62 times (*p* = 0.036) and 1.62 times (*p* = 0.001) higher than that in the patients with single AFLD, respectively (Figure 3, Supplementary Table S4). Moreover, the patients with AFLD&MAFLD had a higher risk of acute (AOR = 1.22, 95% CI: 1.07–1.39, *p* < 0.001) and chronic kidney failures (AOR = 1.65, 95% CI: 1.09–2.51, *p* = 0.018) than the patients with single AFLD. However, no differences in acute and chronic respiratory and hepatic failures were found between the two groups. (Figure 4, Supplementary Table S4).

### 3.3. Characteristics of Length of Stay, Hospital Total Charges, Nonelective Admission, and All-Cause Mortality

The average length of stay was 5.38 ± 6.24 days. The patients with AFLD&MAFLD had a longer length of stay than the patients with single AFLD (5.59 ± 6.60 vs. 5.27 ± 6.05 days, *p* = 0.026). When we selected the length of hospital stay ≥9 days as one of the outcomes to analyze, the patients with AFLD&MAFLD were more likely to stay in hospital ≥9 days (AOR = 1.16, 95% CI 1.02–1.31, *p* = 0.020) than the patients with single AFLD. The patients in the AFLD&MAFLD group cost much more than the single AFLD group. The hospital total charges (in dollars) were higher in the AFLD &MAFLD group than in the single AFLD group (56,820 ± 79,210 vs. 49,840 ± 75,650, *p* < 0.001). The risk of hospital total charges ≥$50,000 was significantly higher in the AFLD&MAFLD group than in the single AFLD group (AOR = 1.12, 95%CI 1.02–1.23, *p* = 0.024). Moreover, the patients with AFLD&MAFLD had a higher risk of nonelective admission than the patients with single AFLD (AOR = 1.28, 95%CI 1.04–1.57, *p* = 0.019). However, there was no significant difference in in-hospital all-cause mortality between these two groups (AOR = 1.003, 95%CI 0.64–1.52, *p* = 0.989, Table 2).

## 4. Discussion

In this study, the patients with AFLD&MAFLD had a higher risk of coronary atherosclerosis, acute myocardial infarction, arrhythmias, asthma, COPD, CKD, and acute and chronic heart and kidney failures than the patients with single AFLD. Moreover, the patients with AFLD&MAFLD had longer hospital stays, higher hospital costs, and higher risk of nonelective admission than the patients with single AFLD, while in-hospital all-cause mortality did not differ significantly between these two groups.

According to the definition, MAFLD was diagnosed when hepatic steatosis was combined with at least one of the following conditions: overweight or obesity, presence of T2DM, or evidence of metabolic dysregulation. In our study, we found more individuals with overweight or obesity, diabetes, hypertension, and hyperlipidemia in the AFLD&MAFLD group than in the single AFLD group, which might lead to more comorbidities and organ failures. Previous studies showed that overweight and obese patients were more likely to have multiple systemic comorbidities than normal-weight individuals [17]. Obesity, metabolic syndrome (MetS), and alcohol intake have synergistic effects on the risk of future liver disease. In the presence of metabolic risk factors, moderate alcohol consumption appears to be significantly more damaging to the liver [18,19]. Obese individuals were more than twice as likely to have multimorbidity as non-obese individuals [20]. The percentage of patients with multimorbidity increased as the rank of obesity increased, increasing most substantially with the progression from overweight to class 1 obesity [21]. Obesity-related infiltration of immune cells, inflammation, and increased oxidative stress promoted metabolic damage in insulin-sensitive tissues, ultimately leading to insulin resistance, organ failure, and premature aging [22]. Moreover, the differences in metabolic status might also contribute to the differences in the risk of comorbidities. Previous studies found that hyperglycemia, hypertension, and hyperlipidemia aggravated systemic insulin resistance, inflammation, and oxidative stress, which can damage the circulatory system [23,24,25], gastrointestinal tract [26,27,28,29], pancreatic β-cells [30,31], liver [32,33], skeletal muscle [32,33], and other system functions [34,35]. The treatment of AFLD at an early stage is crucial for patients. According to the pathological features of alcoholic fatty liver disease, appropriate treatment in an early stage can reverse the fatty infiltration of the liver [36]. In this study, we also found that the patients with AFLD&MAFLD had a longer length of stay and higher hospital total charges than the patients with single AFLD. In addition, the patients with AFLD&MAFLD had a higher risk of nonelective admission than the patients with single AFLD. As discussed earlier, the patients with AFLD&MAFLD were older, and they had more complex comorbidities and organ failures. Thus, these factors might result in more intricate conditions during hospitalization among the patients with AFLD&MAFLD, which might need more time to recover and cost a huge amount of money.

This study was novel in its comparison of the clinical manifestations between the dual-etiology group (AFLD&MAFLD) and the single AFLD group based on the new MAFLD criteria using the NIS database. We found that the establishment of “positive” diagnostic criteria for MAFLD not only stratifies the clinical manifestations of patients with NAFLD [12], but also stratifies the clinical manifestations of patients with AFLD because the diagnostic criteria do not need to exclude alcohol consumption, and this classification method is more conducive to clinical diagnosis and treatment. We also acknowledged certain limitations of this study. First, we were unable to assess the dynamic changes in AFLD&MAFLD status longitudinally as the NIS database does not consider follow-up anthropometry, laboratory tests, and hepatic ultrasound. Second, the diagnostic criteria for MAFLD are still at the stage of recommendation by the expert panel and may change in the future. Although metabolic risk abnormalities, such as obesity, diabetes, hypertension, and hyperlipidemia, are included in the definition of MAFLD, the significant prevalence of these variables in patients with AFLD might impact all-cause and cause-specific mortality. Therefore, the evaluation of the outcomes in the patients with AFLD&MAFLD and single AFLD was complex and influenced by the presence or absence of metabolic risk factors resulting from a spectrum of heterogeneous observations. Finally, the NIS database is a national inpatient database with no long-term follow-up data. In this study, we only evaluated the short-term in-hospital mortality risk for the patients with AFLD&MAFLD and single AFLD. Future studies should evaluate the differences in the risk of long-term mortality between patients with AFLD&MAFLD and single AFLD using cohorts with long-term follow-up data.

## 5. Conclusions

In conclusion, the patients with dual-etiology fatty liver (AFLD&MAFLD) are more likely to have more comorbidities and organ failures compared to the patients with single AFLD. Moreover, the patients with AFLD&MAFLD have longer hospital stays and higher hospital costs than the patients with single AFLD. In the process of diagnosis and treatment of patients with AFLD, “positive” diagnostic criteria of MAFLD should be introduced, and clinicians should pay more attention to patients with AFLD&MAFLD, to reduce the occurrence of comorbidities and organ failures, as well as the burden of AFLD.

## Figures and Tables

**Table 1:** Demographic and clinical characteristics of study participants.

Characteristics	Overall (n = 9269)	Single AFLD Group (n = 6139)	AFLD&MAFLD Group (n = 3130)	*p* Value *
Age (years) (mean ± SD)	49.40 ± 13.0	47.69 ±12.92	52.74 ± 12.42	<0.001
Male (%)	6476 (69.9)	4154 (67.7)	2322 (74.2)	<0.001
Race				<0.001
White (%)	6389 (70.0)	4317 (70.1)	2072 (68.4)	
African American (%)	1066 (11.9)	703 (11.8)	363 (12)	
Hispanic (%)	1170 (13)	750 (12.6)	420 (13.9)	
Asian/Pacific Islander (%)	110 (1.2)	64 (1.1)	46 (1.5)	
Native American (%)	124 (1.4)	77 (1.3)	47 (1.6)	
Other races (%)	262 (2.9)	180 (3.0)	82 (2.7)	
Tobacco abuse (%)	488 (5.3)	336 (5.5)	152 (4.9)	0.208
Overweight (%)	263 (2.8)	0 (0.0)	263 (8.4)	<0.001
Obesity (%)	1239(13.4)	0 (0.0)	1239 (39.6)	<0.001
Hypertension (%)	4305 (46.5)	2240 (36.5)	2065 (66.0)	<0.001
Comorbidity				
Prediabetes (%)	1544 (16.7)	60 (1.0)	1484 (47.4)	<0.001
DM				
T1DM (%)	71 (0.8)	31 (0.5)	40 (1.3)	<0.001
T2DM (%)	1328 (14.3)	0 (0.0)	1328 (42.4)	<0.001
Dyslipidemia				
Hyperlipidemia (%)	1714 (18.5)	321 (5.2)	1393 (44.5)	<0.001
Hypercholesterolemia (%)	2085 (22.5)	533 (8.7)	1552 (49.6)	<0.001
Hypertriglyceridemia (%)	256 (2.8)	121 (2.0)	135 (4.3)	<0.001
Coronary atherosclerosis (%)	759 (8.2)	340 (5.3)	419 (13.4)	<0.001
Myocardial infarction (%)	138 (1.5)	67 (1.1)	71 (2.3)	<0.001
Cerebrovascular disease (%)	93 (1.0)	52 (0.8)	41 (1.3)	0.034
Peripheral vascular disease (%)	110 (1.1)	52 (0.8)	48 (1.5)	0.002
Arrhythmia (%)	1133 (12.2)	620 (10.1)	513 (16.4)	<0.001
Asthma (%)	608 (6.6)	370 (6.0)	238 (7.6)	0.030
COPD (%)	1234 (13.3)	718 (11.7)	516 (16.5)	<0.001
Chronic kidney disease (%)	434 (4.7)	214 (3.5)	220 (7.0)	<0.001
Organ failure				
Acute heart failure (%)	280 (3.0)	144 (2.3)	136 (4.3)	<0.001
Chronic heart failure (%)	237 (2.6)	135 (2.2)	103 (3.3)	0.002
Acute respiratory failure (%)	664 (7.16)	415 (6.76)	249 (7.96)	0.035
Chronic respiratory failure (%)	40 (0.4)	19 (0.3)	21 (0.7)	0.012
Acute kidney failure (%)	1421 (15.33)	819 (13.34)	602 (19.23)	<0.001
Chronic kidney failure (%)	104 (1.1)	52 (0.8)	52 (1.7)	<0.001
Acute hepatic failure (%)	120 (1.29)	69 (1.12)	51 (1.63)	0.42
Chronic hepatic failure (%)	11 (0.1)	5 (0.1)	6 (0.1)	0.145
Ventilator support during operation (%)	451 (4.87)	289 (4.71)	162 (5.18)	0.322
Length of stay (days) (mean ± SD)	5.38 ± 6.24	5.27 ± 6.05	5.59 ± 6.60	0.026
Total Charge (1000 dollars) (mean ± SD)	52.19 ± 76.93	49.84 ± 75.65	56.82 ± 79.21	<0.001
Death (%)	106 (1.11)	64 (1.04)	42 (1.34)	0.201

* Significant difference when comparing between the AFLD and AFLD&MAFLD groups.

**Table 2:** Differences in the risk of length of stay (LOS) ≥ 9 days, total charges (TC) ≥ 50,000 dollars, non-elective admission, and all-cause mortality between AFLD&MAFLD and single AFLD.

Variables	Univariable Model		Multivariable Model	
	OR (95% CI)	*p*-Value	OR (95% CI)	*p*-Value
LOS ≥ 9 days				
AFLD	1 (Reference)		1 (Reference)	
AFLD&MAFLD	1.26 (1.12–1.42)	<0.001	1.16(1.02–1.31)	0.020
TC ≥ 50,000 dollars				
AFLD	1 (Reference)		1 (Reference)	
AFLD&MAFLD	1.28(1.17–1.41)	<0.001	1.12(1.02–1.23)	0.024
Non-elective admission				
AFLD	1 (Reference)		1 (Reference)	
AFLD&MAFLD	1.54(1.27–1.87)	<0.001	1.28(1.04–1.57)	0.019
All-cause mortality				
AFLD	1 (Reference)		1 (Reference)	
AFLD&MAFLD	1.29(0.87–1.91)	0.202	1.003(0.664–1.515)	0.989

Adjusted for age, sex, race, and smoking habit.

## Data Availability

This study used data from the Nationwide Inpatient Sample (NIS) database. For details, please contact access@hcup-us.ahrq.gov. All other data are contained in the article, and its supplementary information is available upon reasonable request.

## Supplementary Materials

The following supporting information can be downloaded at https://www.mdpi.com/article/10.3390/diagnostics13030488/s1, Table S1: Diagnostic Codes Used for Including and Excluding Patients; Table S2: Diagnostic Codes Used for Identifying Comorbidities; Table S3: Differences in the risk of comorbidities between AFLD&MAFLD and single AFLD; Table S4: Differences in the risk of organ failures between AFLD&MAFLD and single AFLD.
