# Supplementary material for: Changes in Clinical Manifestations Due to AFLD Retyping Based on the New MAFLD Criteria: An Observational Study Based on the National Inpatient Sample Database
Source: Diagnostics (Basel). 2023 Jan 29;13(3):488. doi: 10.3390/diagnostics13030488 (PMC9914804; doi:10.3390/diagnostics13030488)
Supplement: Supplementary file 1 [file diagnostics-13-00488-s001.zip › diagnostics-2139176-supplementary.pdf]

Article

Changes in Clinical Manifestations Due to AFLD Retyping Based on the New MAFLD Criteria: An Observational Study Based on the National Inpatient Sample Database

Xiaoshan Feng [1,2,3,4,5,†], Ruirui Xuan [1,2,3,4,5,†], Yingchun Dong [1,2,3,4,5], Xiaoqin Wu [6], Yiping Cheng [1,2,3,4,5], Zinuo Yuan [1,2,3,4,5], Hang Dong [1,2,3,4,5], Junming Han [1,2,3,4,5], Fang Zhong [1,2,3,4,5], Jiajun Zhao [1,2,3,4,5,*] and Xiude Fan [1,2,3,4,5,*]

[1] Department of Endocrinology, Shandong Provincial Hospital, Shandong University, Jinan 250021, China
[2] Shandong Clinical Research Center of Diabetes and Metabolic Diseases, Jinan 250021, China
[3] Shandong Institute of Endocrine and Metabolic Diseases, Jinan 250021, China
[4] Shandong Engineering Laboratory of Prevention and Control for Endocrine and Metabolic Diseases, Jinan 250021, China
[5] Shandong Engineering Research Center of Stem Cell and Gene Therapy for Endocrine and Metabolic Diseases, Jinan 250021, China
[6] Northern Ohio Alcohol Center, Department of Inflammation and Immunity, Cleveland Clinic, Cleveland 44196, OH, USA
* Correspondence: jjzhao@sdu.edu.cn (J.Z.); Tel.: +86-0531-68776049(J.Z.); fanxiudexjtu@163.com (X.F.); Tel.: +86-13186067538 (X.F.)
† These authors contributed equally to this work.

Supplementary Table S1. Diagnostic Codes Used for Including and Excluding Patients

Diagnoses	ICD-10 Codes
**Alcoholic fatty liver disease**	K700
**Hepatic cirrhosis**	K7031, K7031, K717, K7460, K7469, P7881
**Hepatic carcinoma**	C220, C221, C227
**Virus hepatitis**	A5145, B0081, B150, B159, B160, B161, B162, B169, B170, B1710, B1711, B172, B178, B179, B180, B181, B182, B188, B189, B190, B1910, B1911, B1920, B1921, B199, B251, B2681, B581, B942
**Toxic hepatitis**	K712, K713, K714, K7150, K7151, K716
**Autoimmune hepatitis**	K754
**Wilson's disease**	E8301
**Liver cancer**	C220, C221, C222, C223, C224, C227, C228, C229
**Pregnancy**	Z331,Z332,Z333,Z3400,Z3401,Z3402,Z3403,Z3480,Z3481,Z3482,Z3483,Z3491,Z3492,Z3493,Z36,Z360,Z361,Z362,Z363,Z364,Z365,Z3681,Z3682,Z3683,Z3684,Z3685,Z3686,Z3687,Z3688,Z3689,Z368A,Z369,Z3A00,Z3A01,Z3A08,Z3A09,Z3A10,Z3A11,Z3A12,Z3A13,Z3A14,Z3A15,Z3A16,Z3A17,Z3A19,Z3A18,Z3A20,Z3A21,Z3A22,Z3A23,Z3A24,Z3A25,Z3A26,Z3A27,Z3A28,Z3A29,Z3A30,Z3A31,Z3A32,Z3A33,Z3A34,Z3A35,Z3A36,Z3A37,Z3A38,Z3A39,Z3A40,Z3A41,Z3A42,Z3A49,O000,O0000,O0001

ICD, International Classification of Disease.

Supplementary Table S2 Diagnostic Codes Used for Identifying Comorbidities

Diagnoses	ICD-10 Codes
**Myocardial infarction**	I2101, I2102, I2109, I2111, I2119, I2121, I2129, I213, I214, I219, I21A1, I21A9, I220, I221, I222, I228, I229, I252
**Coronary atherosclerosis**	I2583, I2584
**Peripheral vascular disease**	I700, I701, I70201, I70202, I70203, I70208, I70209, I70211, I70212, I70213, I70218, I70219, I70221, I70222, I70223, I70228, I70229, I70231, I70232, I70233, I70234, I70235, I70238, I70239, I70241, I70242, I70243, I70244, I70245, I70248, I70249, I7025, I70261, I70262, I70263, I70268, I70269, I70291, I70292, I70293, I70298, I70299, I70301, I70302, I70303, I70308, I70309, I70311, I70312, I70313, I70318, I70319, I70321, I70322, I70323, I70328, I70329, I70331, I70332, I70333, I70334, I70335, I70338, I70339, I70341, I70342, I70343, I70344 I70345, I70348, I70349, I7035, I70361, I70362, I70363, I70368, I70369, I70391, I70392, I70393, I70398, I70399, I70401, I70402, I70403, I70408, I70409, I70411, I70412, I70413, I70418, I70419, I70421, I70422, I70423, I70428, I70429, I70431, I70432, I70433, I70434, I70435, I70438, I70439, I70441, I70442, I70443, I70444, I70445, I70448, I70449, I7045, I70461, I70462, I70463, I70468, I70469, I70491, I70492, I70493, I70498, I70499, I70501, I70502, I70503, I70508, I70509, I70511, I70512, I70513, I70518, I70519, I70521, I70522, I70523, I70528, I70529, I70531, I70532, I70533, I70534, I70535, I70538, I70539, I70541, I70542, I70543, I70544, I70545, I70548, I70549, I7055, I70561, I70562, I70563, I70568, I70569, I70591, I70592, I70593, I70598, I70599, I70601, I70602, I70603, I70608, I70609, I70611, I70612, I70613, I70618, I70619, I70621, I70622, I70623, I70628, I70629, I70631, I70632, I70633, I70634, I70635, I70638, I70639, I70641, I70642, I70643, I70644, I70645, I70648, I70649, I7065, I70661, I70662, I70663, I70668, I70669, I70691, I70692, I70693, I70698, I70699, I70701, I70702, I70703, I70708, I70709, I70711, I70712, I70713, I70718, I70719, I70721, I70722, I70723, I70728, I70729, I70731, I70732, I70733, I70734, I70735, I70738, I70739, I70741, I70742, I70743, I70744, I70745, I70748, I70749, I7075, I70761, I70762, I70763, I70768, I70769, I70791, I70792, I70793, I70798, I70799, I708, I7090, I7091, I7092, I7100, I7101, I7102, I7103, I711, I712, I713, I714, I715, I716, I718, I719, I720, I721, I722, I723, I724, I725, I726, I728, I729, I7300, I7301, I731, I7381, I7389, I739, I7401, I7409, I7410, I7411, I7419, I742, I743, I744, I745, I748, I749, I770, I771, I772, I773, I774, I775, I776, I7770, I7771, I7772, I7773, I7774, I7775, I7776, I7777, I7779, I77810, I77811, I77812, I77819, I7789, I779

**Cerebrovascular disease**	I6000, I6001, I6002, I6010, I6011, I6012, I602, I6020, I6021, I6022, I6030, I6031, I6032, I604, I6050, I6051, I6052, I606, I607, I608, I609, I610, I611, I612, I613, I614, I615, I616, I618, I619, I6200, I6201, I6202, I6203, I621, I629, I6300, I63011, I63012, I63013, I63019, I6302, I63031, I63032, I63033, I63039, I6309, I6310, I63111, I63112, I63113, I63119, I6312, I63131, I63132, I63133, I63139, I6319, I6320, I63211, I63212, I63213, I63219, I6322, I63231, I63232, I63233, I63239, I6329, I6330, I63311, I63312, I63313, I63319, I63321, I63322, I63323, I63329, I63331, I63332, I63333, I63339, I63341, I63342, I63343, I63349, I6339, I6340, I63411, I63412, I63413, I63419, I63421, I63422, I63423, I63429, I63431, I63432, I63433, I63439, I63441, I63442, I63443, I63449, I6349, I6350, I63511, I63512, I63513, I63519, I63521, I63522, I63523, I63529, I63531, I63532, I63533, I63539, I63541, I63542, I63543, I63549, I6359, I636, I638, I6381, I6389, I639, I6501, I6502, I6503, I6509, I651, I6521, I6522, I6523, I6529, I658, I659, I6601, I6602, I6603, I6609, I6611, I6612, I6613, I6619, I6621, I6622, I6623, I6629, I663, I668, I669, I670, I671, I672, I673, I674, I675, I676, I677, I6781, I6782, I6783, I67841, I67848, I67850, I67858, I6789, I679, I680, I682, I688, I6900, I6901, I69010, I69011, I69012, I69013, I69014, I69015, I69018, I69019, I69020, I69021, I69022, I69023, I69028, I69031, I69032, I69033, I69034, I69039, I69041, I69042, I69043, I69044, I69049, I69051, I69052, I69053, I69054, I69059, I69061, I69062, I69063, I69064, I69065, I69069, I69090, I69091, I69092, I69093, I69098, I6910, I6911, I69110, I69111, I69112, I69113, I69114, I69115, I69118, I69119, I69120, I69121, I69122, I69123, I69128, I69131, I69132, I69133, I69134, I69139, I69141, I69142, I69143, I69144, I69149, I69151, I69152, I69153, I69154, I69159, I69161, I69162, I69163, I69164,

	I69165, I69169, I69190, I69191, I69192, I69193, I69198, I6920, I6921, I69210, I69211, I69212, I69213, I69214, I69215, I69218, I69219, I69220, I69221, I69222, I69223, I69228, I69231, I69232, I69233, I69234, I69239, I69241, I69242, I69243, I69244, I69249, I69251, I69252, I69253, I69254, I69259, I69261, I69262, I69263, I69264, I69265, I69269, I69290, I69291, I69292, I69293, I69298, I6930, I6931, I69310, I69311, I69312, I69313, I69314, I69315, I69318, I69319, I69320, I69321, I69322, I69323, I69328, I69331, I69332, I69333, I69334, I69339, I69341, I69342, I69343, I69344, I69349, I69351, I69352, I69353, I69354, I69359, I69361, I69362, I69363, I69364, I69365, I69369, I69390, I69391, I69392, I69393, I69398, I6980, I6981, I69810, I69811, I69812, I69813, I69814, I69815, I69818, I69819, I69820, I69821, I69822, I69823, I69828, I69831, I69832, I69833, I69834, I69839, I69841, I69842, I69843, I69844, I69849, I69851, I69852, I69853, I69854, I69859, I69861, I69862, I69863, I69864, I69865, I69869, I69890, I69891, I69892, I69893, I69898, I6990, I6991, I69910, I69911, I69912, I69913, I69914, I69915, I69918, I69919, I69920, I69921, I69922, I69923, I69928, I69931, I69932, I69933, I69934, I69939, I69941, I69942, I69943, I69944, I69949, I69951, I69952, I69953, I69954, I69959, I69961, I69962, I69963, I69964, I69965, I69969, I69990, I69991, I69992, I69993, I69998, G450, G451, G452, G453, G454, G458, G459, G460, G461, G462, G463, G464, G465, G466, G467, G468
**Arrhythmia**	I470, I471, I472, I479, I480, I481, I4811, I4819, I482, I4820, I4821, I483, I484, I4891, I4892, I491, I492, I493, I4940, I4949, I495, I498, I499
**Chronic obstructive pulmonary disease**	J40, J410, J411, J418, J42, J430, J431, J432, J438, J439, J440, J441, J449, J4520, J4521, J4522, J4530, J4531, J4532, J4540, J4541, J4542, J4550, J4551, J4552, J45901, J45902, J45909, J45990, J45991, J45998, J470, J471, J479
**Asthma**	J4520, J4521, J4522, J4530, J4531, J4532, J4540, J4541, J4542, J4550, J4551, J4552, J45901, J45902, J45909, J45990,J45991,J45998
**Chronic respiratory failure**	J9610, J9611, J9612
**Acute respiratory failure**	J80, J95821, J95822, J9600, J9601, J9602
**Acute hepatic failure**	K7040, K7041, K7200, K7201
**Chronic hepatic failure**	K7210, K7211
**Chronic kidney disease**	N181, N182, N183, N1830, N1831, N1832, N184, N185, N186, N189, R880
**Chronic kidney failure**	N1830, N1831, N1832, N184, N185, N186

**Acute kidney failure**	N170, N171, N172, N178, N179, N19
**Overweight**	Z6825, Z6826, Z6827, Z6828, Z6829, E663, DE660A, E669O
**Obesity （grade 1-3）**	Z6830, Z6831, Z6832, Z6833, Z6834, DE660B， Z6835, Z6836, Z6837, Z6838, Z6839, DE660C， Z6841, Z6842, Z6843, Z6844, Z6845, E6601, E662, DE660E, DE660F, DE660G, DE660H
**Hyperlipidemia**	**Hypercholesteremia**<br>E780, E7800, E7801
	**Hypertriglyceridemia**<br>E781
	**Hyperlipemia**<br>E782, E784, E7849, E785, E783, E7841
**Hypertension**	H35031, H35032, H35033, H35039, G932, I10, I110, I119, I120, I129, I130, I1310, I1311, I132, I150, I151, I152, I158, I159, I160, I161, I169, I674, R030, I973
**Hyperglycemia**	**Prediabetes**<br>R7301, R7302, R7303
	**T1DM**
	E108, E109, E1010, E1011, E1021, E1022, E1029, E10311, E10319, E10321, E103213, E103212, E103211, E103219, E10329, E103293, E103292, E103291, E103299, E10331, E103313, E103312, E103311, E103319, E10339, E103393, E103392, E103391, E103399, E10341, E103413, E103412, E103411, E103419, E10349, E103493, E103492, E103491, E103499, E10351, E103513, E103512, E103511, E103519, E103523, E103522, E103521, E103529, E103533, E103532, E103531, E103539, E103543, E103542, E103541, E103549, E103553, E103552, E103551, E103559, E10359, E103593, E103592, E103591, E103599, E1036, E1039, E1037X3, E1037X2, E1037X1, E1037X9, E1040, E1041, E1042, E1044, E1043, E1049, E1051, E1052, E1059, E10610, E10618, E10620, E10621, E10622, E10628, E10630, E10638, E10641, E10649,<br>E1065, E1069
	**T2DM**

	E1100, E1101, E1111, E1110, E1122, E1121, E1129, E11311, E11319, E11321, E113213, E113212, E113211, E113219, E11329, E113293, E113292, E113291, E113299, E11331, E113313, E113312, E113311, E113319, E11339, E113393, E113392, E113391, E113399, E11341, E113413, E113412, E113411, E113419, E11349, E113493, E113492, E113491, E113499, E11351, E113513, E113512, E113511, E113519, E113523, E113522, E113521, E113529, E113533, E113532, E113531, E113539, E113543, E113542, E113541, E113549, E113553, E113552, E113551, E113559, E11359, E113593, E113592, E113591, E113599, E1136, E1139, E1144, E1143, E1141, E1140, E1142, E1149, E1152, E1151, E1159, E11620, E11621, E11622, E11628, E11610, E11618, E11630, E11638, E1137X3, E1137X2, E1137X1, E1137X9, E11641, E11649, E1165, E1169, E118, E119

ICD, International Classification of Diseases; T1DM, Type 1 diabetes mellitus; T2DM, Type 2 diabetes mellitus.

Supplementary Table S3. Differences in the risk of comorbidities between AFLD&MAFLD and single AFLD.

Variables	Univariable model		Multivariable model	
	OR (95% CI)	*P-value*	OR (95% CI)	*P-value*
Myocardial infarction				
AFLD	1 (Reference)		1 (Reference)	
AFLD&MAFLD	2.10 (1.50-2.95)	<0.001	1.51 (1.06-2.16)	0.022
Coronary atherosclerosis				
AFLD	1 (Reference)		1 (Reference)	
AFLD&MAFLD	2.64 (2.27-3.06)	<0.001	1.99(1.70-2.35)	<0.001
Cerebrovascular disease				
AFLD	1 (Reference)		1 (Reference)	
AFLD&MAFLD	1.55(1.03-2.35)	0.034	1.22(0.80-1.86)	0.365
Arrhythmia				
AFLD	1 (Reference)		1 (Reference)	
AFLD&MAFLD	1.37(1.20-1.56)	<0.001	1.33(1.16-1.52)	<0.001
Asthma				
AFLD	1 (Reference)		1 (Reference)	
AFLD&MAFLD	1.40(1.17-1.68)	<0.001	1.40(1.17-1.68)	<0.001
COPD				
AFLD	1 (Reference)		1 (Reference)	
AFLD&MAFLD	1.49 (1.32-1.68)	<0.001	1.19 (1.02-1.38)	0.023
CKD				
AFLD	1 (Reference)		1 (Reference)	
AFLD&MAFLD	1.61(1.31-1.97)	<0.001	1.36(1.06-1.74)	0.014
Peripheral vascular disease				
AFLD	1 (Reference)		1 (Reference)	
AFLD&MAFLD	1.82(1.23-2.71)	0.002	1.26(0.83-1.89)	0.209

Adjusted for age, sex, race, smoking habit, and other comorbidities correspondingly.
Abbreviation: AFLD, alcoholic fatty liver disease; AFLD&MAFLD, alcoholic fatty liver disease and metabolic-associated fatty liver disease (dual-etiology fatty liver disease); COPD, chronic obstructive pulmonary disease; CKD, chronic kidney disease; OR, odds ratio.

Supplementary Table S4. Differences in the risk of organ failures between AFLD&MAFLD and single AFLD.

Variables	Univariable model		Multivariable model	
	OR (95% CI)	*P-value*	AORs (95% CI)	*P-value*
Acute heart failure				
AFLD	1 (Reference)		1 (Reference)	
AFLD&MAFLD	1.89 (1.49-2.40)	<0.001	1.62(1.03-2.53)	0.036
Chronic heart failure				
AFLD	1 (Reference)		1 (Reference)	
AFLD&MAFLD	1.50(1.15-1.95)	0.002	1.62(1.21-2.18)	0.001
Acute respiratory failure				
AFLD	1 (Reference)		1 (Reference)	
AFLD&MAFLD	1.19 (1.01-1.40)	0.004	1.09 (0.91-1.31)	0.361
Chronic respiratory failure				
AFLD	1 (Reference)		1 (Reference)	
AFLD&MAFLD	2.18 (1.17-4.05)	0.012	1.88(0.94-3.73)	0.074
Acute kidney failure				
AFLD	1 (Reference)		1 (Reference)	
AFLD&MAFLD	1.55 (1.38-1.74)	<0.001	1.22 (1.07-1.39)	<0.001
Chronic kidney failure				
AFLD	1 (Reference)		1 (Reference)	
AFLD&MAFLD	1.98(1.34-2.91)	<0.001	1.65(1.09-2.51)	0.018
Acute hepatic failure				
AFLD	1 (Reference)		1 (Reference)	
AFLD&MAFLD	1.48(1.02-2.15)	0.038	1.27(0.867-1.873)	0.218
Chronic hepatic failure				
AFLD	1 (Reference)		1 (Reference)	
AFLD&MAFLD	3.25(0.97-10.93)	0.057	2.34(0.65-8.38)	0.191

Adjusted for age, sex, race, comorbidities, smoking habit, and other organ failures correspondingly.

Abbreviation: AFLD, alcoholic fatty liver disease; AFLD&MAFLD, alcoholic fatty liver disease and metabolic-associated fatty liver disease (dual-etiology fatty liver disease); OR, odds ratio.
